# Synthesis and application of g-C_3_N_4_/Fe_3_O_4_/Ag nanocomposite for the efficient photocatalytic inactivation of *Escherichia coli* and *Bacillus subtilis* bacteria in aqueous solutions

**DOI:** 10.1186/s13568-021-01324-3

**Published:** 2021-12-03

**Authors:** Soudabeh Ghodsi, Ali Esrafili, Hamid Reza Sobhi, Roshanak Rezaei Kalantary, Mitra Gholami, Ramin maleki

**Affiliations:** 1 Department of Environmental Health Engineering, School of Public Health, Isfahan University of Medical Sciences, Isfahan, Iran; 2 Department of Environmental Health Engineering, School of Public Health, Iran University of Medical Sciences, Tehran, Iran; 3 Research Center for Environmental Health Technology, Iran University of Medical Sciences, Tehran, Iran; 4 Department of Chemistry, Payame Noor University, Tehran, Iran

**Keywords:** Photocatalysis, *E. coli*, *B. subtilis*, g-C_3_N_4_/Fe_3_O_4_/Ag, Inactivation

## Abstract

Contamination of water with bacteria is one of the main causes of waterborne diseases. The photocatalytic method on the basis of bacterial inactivation seems to be a suitable disinfectant due to the lack of by-products formation. Herein, g-C_3_N_4_/Fe_3_O_4_/Ag nanocomposite combined with UV-light irradiation was applied for the inactivation two well-known bacteria namely, *E. coli* and *B. subtilis*. The nanocomposite was prepared by a hydrothermal method, and subsequently it was characterized by XRD, FT-IR, SEM, EDX and PL analyses. The optimum conditions established for the inactivation of both bacteria were as follows: nanocomposite dosage 3 g/L and bacterial density of 10^3^ CFU/mL. In the meantime, the efficient inactivation of *E. coli* and *B. subtilis* took 30 and 150 min, respectively. The results also revealed that inactivation rate dropped with an increase in the bacterial density. It is also pointed out that OH˚ was found out to be the main radical species involved in the inactivation process. Finally, the kinetic results indicated that the inactivation of *E. coli* and *B. subtilis* followed the Weibull model. It is concluded that C_3_N_4_/Fe_3_O_4_/Ag nanocomposite along with UV-light irradiation is highly effective in inactivating *E. coli* and *B. subtilis* bacteria in the aqueous solutions.

## Introduction

Given on-going growing population and climate change phenomena, provision of high quality and clean water from reused aqueous sources remains a great challenge (Widi et al. 2018). This highlights the importance of water purification with regard to chemical and microbial contamination (Rojviroon and Sirivithayapakorn, 2018; Feilizadeh et al. 2015). It is crystal clear that the presence of pathogens in water has become a big concern worldwide (Fang et al. 2013). Bacteria, viruses and fungi are widely found in water resources and pose significant health risks to humans and animals (Xia et al. 2017).

Recently, the development of new technologies for the treatment of pathogens in aquatic environments has dramatically increased (Widi et al. 2018). Until now, various methods such as chlorination, UV and ozone have been used to disinfect and remove pathogens (Ouyang et al. 2016). The major drawback of the chlorination process is the reaction of chlorine with the natural organic matter (NOM) present in water which leads to the formation of disinfectionby-products (DBPs) namely, Trihalomethanes (THMs) and Haloacetic acids (HAAs) (Li et al. 2015; Zazouli et al. 2017). These compounds have high carcinogenic effects even at low concentration levels (Zazouli et al. 2017). UV method is effectively used to inactivate microbial agents, but it is expensive and requires high level of energy (Zhang et al. 2014). Moreover, many pathogens are resistant to UV and chlorine (Xia et al. 2017). In recent years, advanced oxidation processes have widely been applied for water and wastewater treatment (Abeledo-Lameiro et al. 2016).

Advanced oxidation processes (AOPs) are founded on the basis of the production of reactive oxygen species (ROSs) (Zhang et al. 2017). The ROSs produced during the photocatalytic process can damage biologically vital macromolecules including DNA, proteins and lipids and alter cell permeability (Wang et al. 2015b).

Heterogeneous catalysts are widely implemented to decompose organic pollutants and inactivate microbial agents and pathogens (Armon et al. 2004). In these catalysts, the electrons lying within the valence band are stimulated and pushed through the conduction band leaving behind a hole in the valence band (Zhang et al. 2017). The main feature of these catalysts are non-toxicity and high stability (Di Palma et al. 2019).

Lately, Carbon nitride graphite (g-C_3_N_4_)-based nanocomposites have extensively been used for the photocatalytic degradation of various pollutants (Wang et al. 2015a). g-C_3_N_4_ is a polymeric organic semiconductor and has properties such as environmental compatibility, high chemical stability, low cost. It is of two-dimensional structure and low-energy band width (2.7 eV) (Mousavi and Habibi-Yangjeh, 2017). However, the rapid re-coupling of electron–hole pairs remains the main problem associated with the use of g-C_3_N_4_ is, which results in the reduction of photocatalytic activity (Mousavi and Habibi-Yangjeh, 2016). As a remedy, metallic/ nonmetallic doping and combination with different semiconductors have been introduced (Pant et al. 2017). In addition, the separation of the catalysts used in the photocatalytic processes is another setback to be overcome (Mousavi and Habibi-Yangjeh, 2017). To fix the above-mentioned problem, the combination of Fe_3_O_4_ nanoparticles with g-C_3_N_4_ sheets has been proposed. This facilitates the quick separation of catalysts from the refined solutions using an external magnet (Akhundi and Habibi-Yangjeh, 2017). A number of studies have shown that g-C_3_N_4_/Fe_3_O_4_ can improve the performance of photocatalytic processes (Ding et al. 2018). It should also be noted that Fe_3_O_4_ nanoparticles can act as intermediates for the rapid transfer of producing electrons due to their high conductivity (Li et al. 2019). Thus, it seems that the introduction of highly conductive elements within Fe_3_O_4_ nanoparticles could certainly improves the separation efficiency of charge carriers (Ghodsi et al. 2020). On the other hand, loading semiconductor surfaces with metals such as Pt, Au and Ag can enhance the photocatalytic activity under light irradiation (Mousavi and Habibi-Yangjeh, 2015).

Amongst the mentioned conductive metals, the disinfection properties of Ag have well been understood for a long time. The advances in nanotechnology have also improved the efficiency of its disinfection. On the other hand, Ag is not associated with the production of any by-products, nor the creation of odor, taste, color, etc. As well as being highly effective in disinfection. Ag is non-toxic, non-irritating, non-allergic, hydrophilic, tolerant to various conditions (ie., very stable), environment friendly, heat resistant and does not escalate the resistance and adaptability of microorganisms (Ma et al. 2016; Tran and Le, 2013; Sondi and Salopek-Sondi, 2004).

Briefly, in this study, g-C_3_N_4_/Fe_3_O_4_/Ag nanocomposite was initially synthesized and characterized by respective hydrothermal and SEM, EDX, XRD, FT-IR and PL methods. The applied nanocomposite was used to inactivate the target bacteria. The Gram-positive bacterium (*Bacillus subtilis ATCC 6636*) and the Gram-negative bacterium (*Escherichia coli ATCC 25922*) were used as the target models throughout. Following on, the Weibull, Log-Linear, and Biphasic models were also used to describe the kinetic behavior of the bacterial inactivation.

## Experimental

### Materials

Melamine (C_3_H_6_N_6_, 99%), silver nitrate (AgNO_3_, 99.8%), iron chloride tetra hydrate (FeCl_2_.4H_2_O, 98%),, Polyvinylpyrrolidone (PVP, 99%), tert-butanol (C_4_H_10_O), ammonium oxalate (C_2_H_8_N_2_O_4_), benzoquinone (C_2_H_4_O_3_), sodium hydroxide (NaOH), hydrochloric acid (HCl, 99%), ethanol (C_2_H_5_OH, 98%), methanol (CH_3_OH, 98%), EMB agar culture medium, BHI culture medium, Loria Bertani (LB) culture medium, glycerol (C_3_H_8_O_3_, 10%), blood agar (BA) culture medium, sulfuric acid (H_2_SO_4_, 98%), Barium chloride (BaCl_2_), ammonia (NH_3_, 28%) were obtained from the Merck Company.

### Synthesis of nanocomposite

Yellow powder g-C_3_N_4_ was obtained by heating melamine in the furnace at 550 ºC for 4 h. To prepare g-C_3_N_4_/Fe_3_O_4_/Ag nanocomposite, a hydrothermal method was implemented. Briefly, 50 g g-C_3_N_4_ was added to 30 mL distilled water and dissolved with aid of ultrasonic waves. After that, 0.5 g of FeCl2.4H2O, 0.025 g of AgNO_3_ and 0.1 g PVP were added to the solution while stirring for 3 h. Then 2.5 mL NH_3_ was added to the above solution and it was vigorously agitated for 10 min. The obtained suspension was transferred to the Teflon cell and autoclaved at 140 °C for 3 h. After that, at room temperature, the suspension was filtered and washed with water and ethanol and subsequently dried at 80 °C for 12 h (Pant et al. 2017).

### Characterization

Following the preparation of g-C_3_N_4_/Fe_3_O_4_/Ag, the physico-chemical properties of the nanocomposite was determined by the identification of the crystalline phase by the XRD (X-ray diffraction) experiments within the range of 2θ = 20–80°. The presence of Fe and Ag elements in the structure of the nanocomposite was confirmed by the energy dispersive X-ray (EDX) analysis. Scanning electron microscopy (SEM) was used to determine the morphology of the synthesized catalyst. Finally, to identify the functional groups of the nanocomposite the Fourier-transform infrared spectroscopy (FT-IR) and photoluminescence (PL) techniques were implemented.

### Preparation of bacterial samples

The bacterial strains used in this study included the Gram-negative bacterium (*Escherichia coli ATCC 25922*) and the Gram-positive bacterium (*Bacillus subtilis ATCC 6636*). The bacteria were lyophilized from the collection center of the Iranian industrial microorganisms. To remove the lyophilized bacteria, each bacterium was inoculated with 1 ml of the BHI liquid medium and incubated for 24 h. Then, the BHI medium containing 10% glycerol was prepared for the long-term storage of the bacteria. Following on, 10 µl of the desired bacteria were individually transferred to the containing-glycerin BHI media and stored at −18 °C. For daily inactivation tests, the standard bacterial strain samples were placed in the incubator to freeze. After that, 0.1 ml of each sample was heated on a shaker at a speed of 180 rpm in the LB culture medium at 37 °C for 18 h. The bacteria were finally separated by centrifugation (5000 rpm, 15 min) and washed with the normal saline water (0.9% w/w) to remove the residual culture medium (Ruales-Lonfat et al. 2016).

**Fig. 1:**
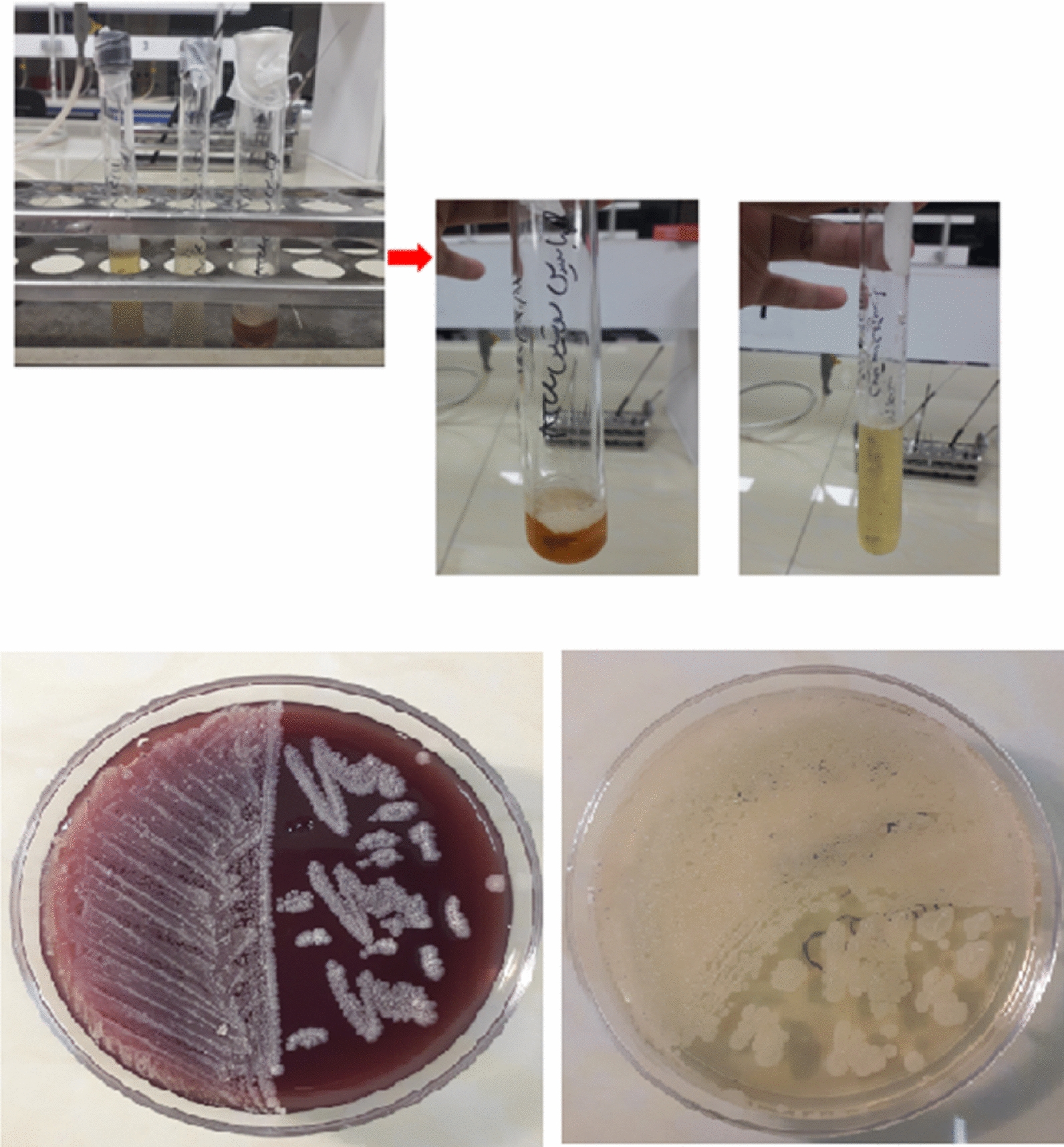
Removal of the bacteria from the lyophilized phase followed by the plate transfer

Figure 1 shows the stages of bacterial extraction from lyophilization phase, in which the bacterium was cultured in TSA and BHI Culture medium after leaving the lyophilization state and finally cultured on the final culture medium to multiply the bacteria.

### Photocatalytic experiments

To perform the photocatalytic disinfection, a number of fixed levels of nanocomposite (0.5, 1.5, 3, 5 g/L) were added in 100 ml sterile salt (0.9% w/w) while stirring by ultrasonic waves (35 kHz) for 1 min (Blatchley et al. 2005). Following that, the specific bacterial densities (*Escherichia coli ATCC 25,922* and *Bacillus subtilis ATCC 6636*) were prepared by an optical density (OD) method and added to the solution (pH 7) and subsequently exposed to the UV lamp (3.3 mw/cm^2^) placed 10 cm above the reactor. At each sampling stage, 100 µL of the diluted sample was added to the culture medium. In a further development, the samples containing the *Escherichia coli* were homogenized on the EMB agar medium and incubated at 37° C for 24 h. The *Bacillus subtilis* sampling was similar to that of *Escherichia coli*, except for the fact that the sample was incubated in the BA culture medium at 30 °C for 24 h. The number of colonies was then counted using the counter colony according to the following Equation (Spuhler et al. 2010; Matin et al. 2018).

1 $$C=\frac{n\times d}{V}$$

C: CFU / mL, n: Number of colonies on a plate, d: Growth Factor, V: Size of transitional sample for culture on plate.

## Kinetic models

### Logarithmic model (log-linear model)

The linear logarithm or the Chick-Watson model is the modified form of the Chick model. The model assumes that there is a stoichiometric relationship between the disinfectant molecules and the number of inactivated microorganisms. The Chick-Watson equation is described as follows:

2 $$Log\frac{{N}_{t}}{{N}_{0}}=-K.C.t\to Log\frac{{N}_{t}}{{N}_{0}}=-{K}_{ap}.t$$

where N_0_ and N_t_ are the microbial density before and after the inactivation process. C, K and t are the concentration of disinfectant, the first-rate inactivation and the inactivation time, respectively (Sun et al. 2007).

### Weibull model

Mafart et al. developed a deactivation kinetic model based on the Weibull distribution (Mafart et al. 2002). Unlike the first-rate model, which assumes the bacterial population is homogenous, Mafart et al. hypothesized that the microbial population would be so heterogeneous that each cell death in the face of external stresses would require different contact times depending on their level of resistance. The cells follow the Weibull distribution model which is expressed as presented below:

3 $$Log\frac{{N}_{t}}{{N}_{0}}=-{\left(\frac{t}{\delta }\right)}^{\beta }$$

δ represents the time required for the first part of the reduction, the duration at which the first logarithmic decline occurs in the bacterial population. β values are varied with the shape of the equation curve. At β > 1, the curve has a downward concave shape whilst at β < 1 an analogous upward shape is observed. At β = 1, the first-order linear logarithm model is seen (Albert and Mafart 2005).

### Biphasic model

This model, which is based on two fractions, was proposed by Cerf in 1977. It is assumed that there are two subpopulation groups with different susceptibility to disinfection.

4 $$Log\frac{{N}_{t}}{{N}_{0}}=Log\left[P.{e}^{{-k}_{1}.t}+\left(1-P\right).{e}^{{-k}_{2}.t}\right]$$

where P represents a fraction of living microorganisms related to the group 1 subpopulation and (1-P) is indicative of a fraction of living microorganisms related to the group 2 subpopulation. K_1_ is the kinetic constant for the sensitive population and K_2_ is the kinetic constant for the higher resistance population (Cerf 1977).

## Results

### Characterization of nanocomposites

**Fig. 2:**
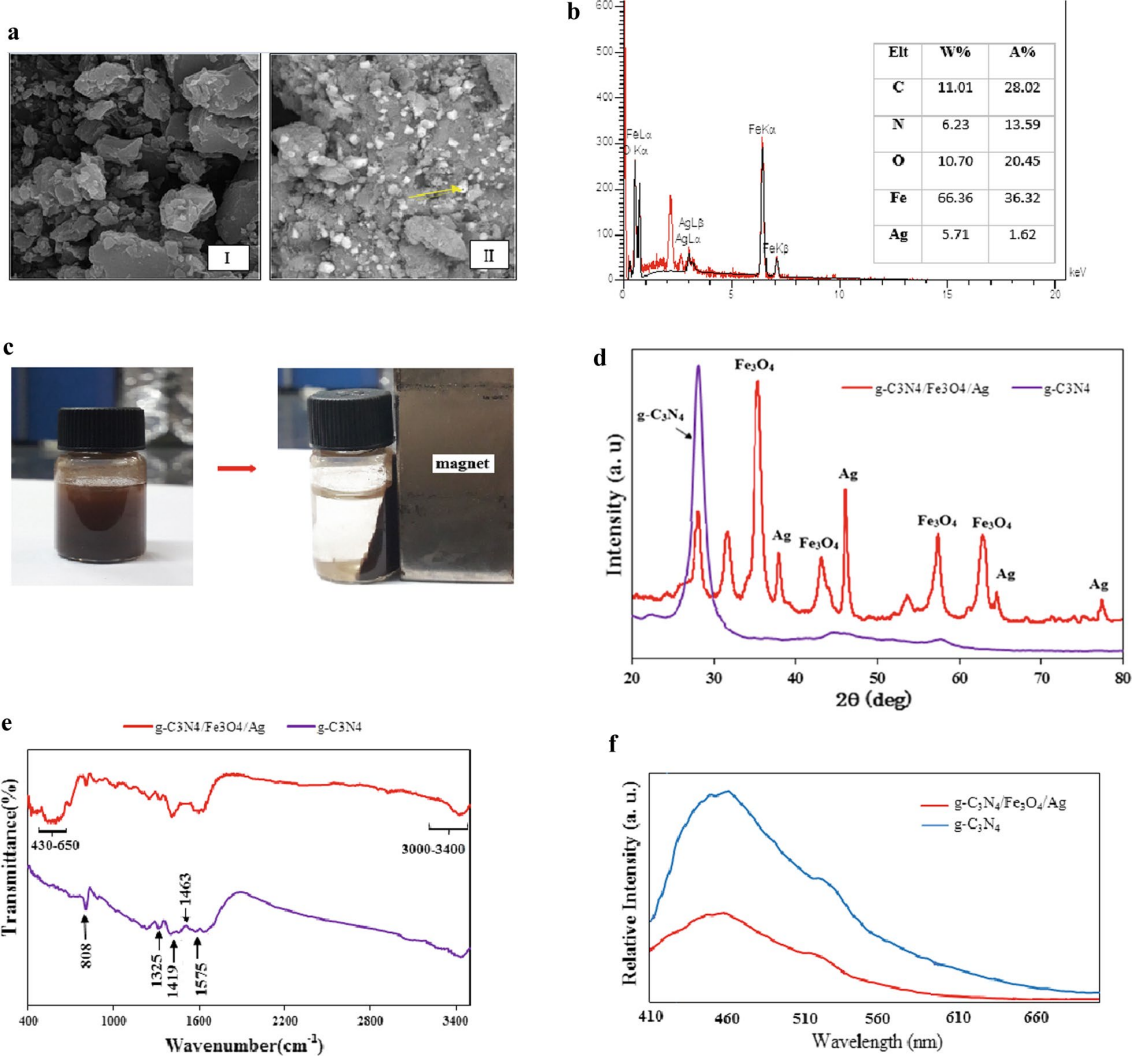
**a** SEM image of pristine g-C_3_N_4_ sheets (I), SEM image of g-C_3_N_4_/Fe_3_O_4_/Ag (II), **b** EDX spectra of g-C_3_N_4_/Fe_3_O_4_/Ag nanocomposite, **c** Magnetism property of C_3_N_4_/Fe_3_O_4_/Ag nanocomposite, **d** XRD images of g-C_3_N_4_ and g-C_3_N_4_/Fe_3_O_4_/Ag, **e** FT-IR images of g-C_3_N_4_ and g-C_3_N_4_/Fe_3_O_4_/Ag, **f** PL Spectra of g-C_3_N_4_ and g-C_3_N_4_/Fe_3_O_4_/Ag

SEM analysis was used to identify the morphology and the composition of the compounds within their surface layers. Figure 2a(I) shows the structure of pure g-C_3_N_4_ with overlapping irregular plate structure. Figure 2a (II) exhibits the structure of g-C_3_N_4_/Fe_3_O_4_/Ag nanocomposite in which the distribution of Fe_3_O_4_/Ag onto g-C_3_N_4_ is marked by the arrow.

EDX analysis was performed to identify the elements present in the nanocomposite structure. The results confirmed the presence of Ag, Fe, O, N and C elements in the nanocomposite structure as well as the fractional weight for each element (Fig. 2b).

The magnetic properties of g-C_3_N_4_/Fe_3_O_4_/Ag nanocomposites is illustrated in Fig. 2c. As can clearly be seen, the nanocomposite was completely separated off the solution phase following the inactivation process underlying a remarkable magnetic propery of g-C_3_N_4_/Fe_3_O_4_/Ag nanocomposite.

The X-ray diffraction (XRD) pattern was used to identify the phase type as well as the crystalline properties of g-C_3_N_4_ and the nanocomposite. The XRD analyses were performed within the 2θ range of 20–80° for pure g-C_3_N_4_ and g-C_3_N_4_/Fe_3_O_4_/Ag nanocomposite. For pure g-C_3_N_4_, a strong peak at 27.6 was observed, which is consistent with the planes (002), referring to an aromatic compound in Fig. 5. In the case of the nanocomposite, the observed peaks were located at 30.2, 35.8, 43.5, 53.7, 57.3 and 62.7°, which are in agreement with the planes (220), (311), (400), (422), (511), (440), respectively. They are all related to Fe_3_O_4_ structure and consistent with the results obtained from the previous studies (Akhundi and Habibi-Yangjeh, 2016; Zhu et al. 2017). Also in the XRD pattern of the nanocomposite, a small drop in the peak intensity compared to that of pure g-C_3_N_4_ could be attributed to the accumulation of Fe_3_O_4_ in the nanocomposite structure. The respective peaks indexed at 38, 44.2, 64.4 and 77.4° refer to the planes (111), (200), (220) and (311) implying the presence of Ag in the nanocomposite structure(Zhu et al. 2016).

In a further related confirmatory analysis, FT-IR was implemented to identify the organic functional groups within the structure of the compounds. Figure 6 represents the FT-IR spectra of pure g-C_3_N_4_ and g-C_3_N_4_/Fe_3_O_4_/Ag at the range of 400–3900 cm^−1^. Within the spectra shown, pure g-C_3_N_4_ is highlighted with a broad absorption band from 3000 to 3400 confirming the tensile (–NH) and (–NH_2_) modes. The peaks located at 1251 cm^−1^, 1325 cm^−1^, 1419 cm^−1^, 1463 cm^−1^, 1575 cm^−1^ and 1639 cm^−1^ are related to (C–N) and (C=N) bonds. In addition, the peak shown at 808 cm^−1^ is related to the s-triazine units and the broad band shown in the far right (430–650 cm^−1)^ is attributed to Fe–O.

It is assumed that transfer and recombination process involving the electron–hole pairs plays an important role in a photocatalytic activity. Moreover, efficient separation of charge carriers can improve the photocatalytic activity. For this reason, photoluminescence (PL) technique was applied to investigate the coupling (i.e., recombination) of the cavities and electrons. The PL spectrum is depicted in Fig. 2f. As can be seen, the strong emission peak is observed for g-C_3_N_4_ compared to that of g-C_3_N_4_/Fe_3_O_4_/Ag. The lower PL intensity is attributed with the favorable electrical conductivity expected for Fe_3_O_4_ and Ag. This brings about an efficient electron transfer from the g-C_3_N_4_ conduction band to the Fe_3_O_4_ and Ag preventing the recombination of the charge carriers followed by an improvement in the photocatalytic activity. It is noted that our PL findings are in agreement with the results published elsewhere (Zhu et al. 2016; Pant et al. 2014).

### The effect of nanocomposite dosage

**Fig. 3:**
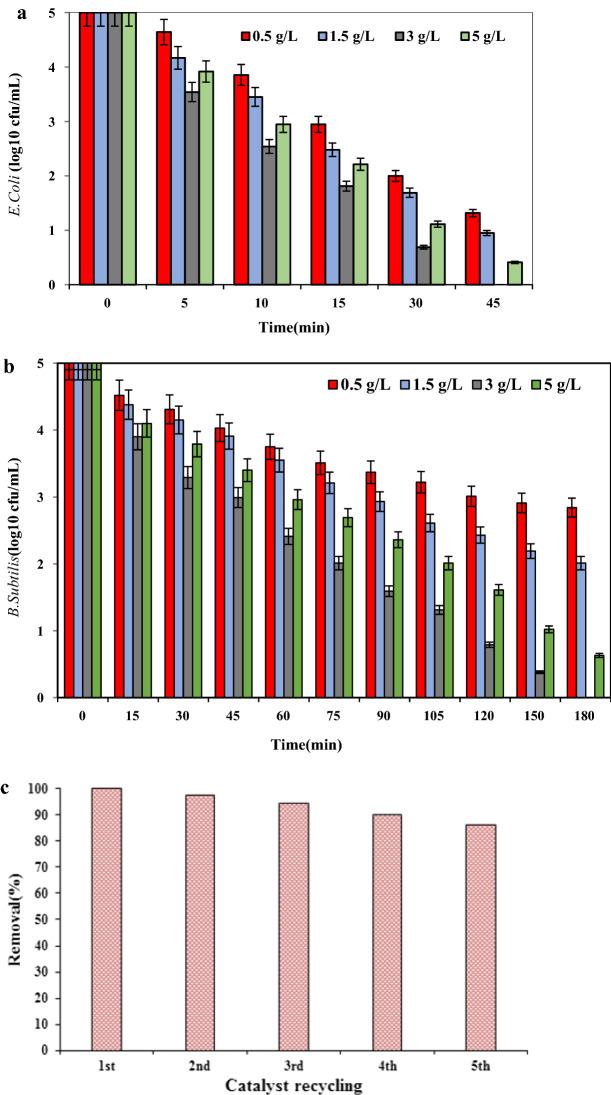
**a** Effect of catalyst dosage on *E. coli* inactivation rate, **b** Effect of catalyst dosage on *B. subtilis* inactivation rate, **c** bacterial inactivation efficiency for 3–5 repeated experiments using recycled sample

The effect of g-C_3_N_4_/Fe_3_O_4_/Ag nanocomposite dosage on the photocatalytic disinfection of the target bacteria was investigated at a specified pH 7. Since the target bacteria (*E. coli* and *B. subtilis*) are sensitive to the change in the environmental conditions, the neutral pH (ca. pH 7) was temporarily chosen. The results exhibiting effect of nanocomposite dosage on the inactivation of the bacteria are illustrated in Fig. 3a, b. It was revealed that the inactivation rate for the target bacteria escalated with the rise in the nanocomposite dosage from 0.5 to 3 g/L. The inactivation rate for *E. coli* increased from 73.6 to 100% at 45 min while the corresponding value jumped for *B. subtilis* from 43.2 to 100% at 180 min.

### The effect of bacterial density on inactivation rate

**Fig. 4:**
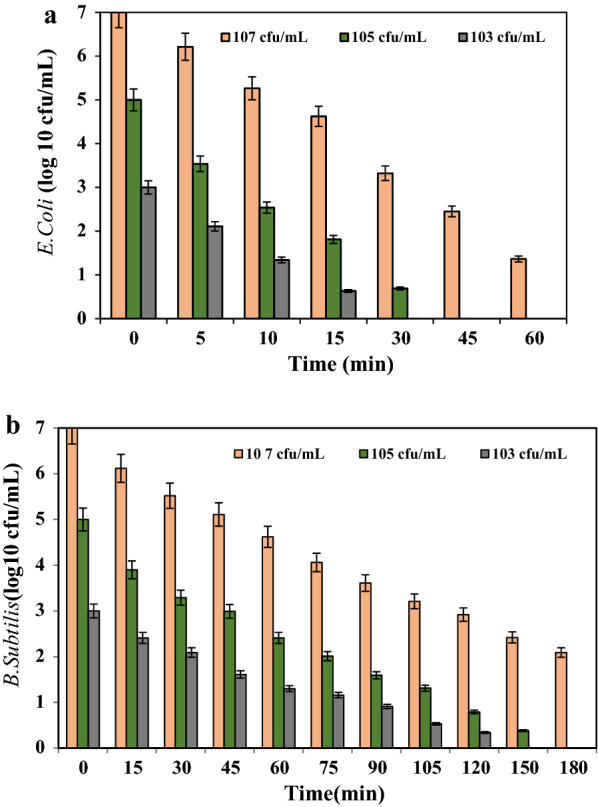
**a** Effect of initial bacterial density on *E. coli* inactivation, **b** Effect of initial bacterial density on *B. subtilis* inactivation

The effect of bacterial density on the inactivation rate of *E.Coli* and *B. subtilis* is depicted in Fig. 4a, b. The results demonstrated that with increasing the bacterial density from 10^3^ to 10^7^ CFU/mL the inactivation rates decreased for both target bacteria over time.

### Effect of irradiation on inactivation of bacteria

**Fig. 5:**
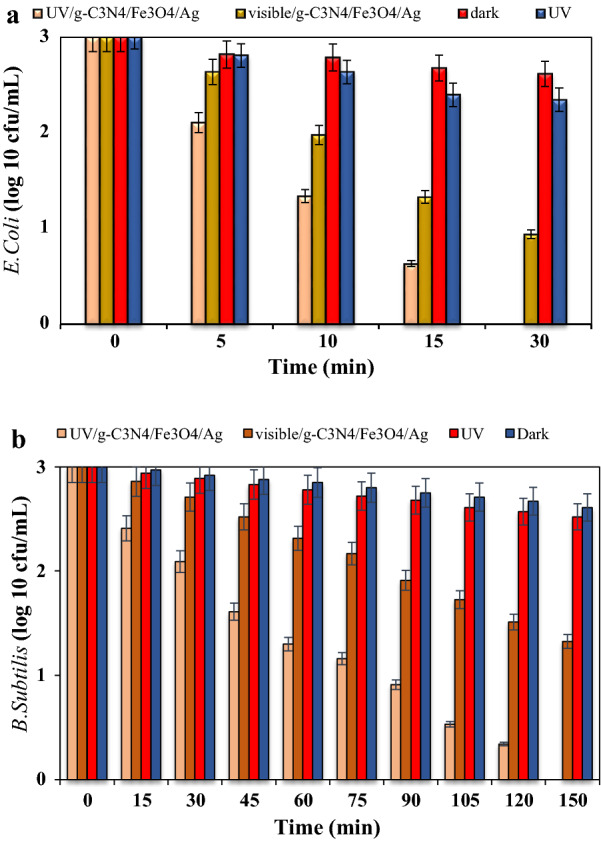
**a** Effect of irradiation on inactivation of *E. coli* inactivation under the optimal conditions, **b** Effect of irradiation on inactivation of *B. subtilis* inactivation under the optimal conditions

In a yet further development, at the presence of the nanocomposite (g-C_3_N_4_/Fe_3_O_4_/Ag) the impact of irradiation on the inactivation rate of the bacteria of interest were thoroughly examined. For this reason, a number of experiments were performed under the dark and UV/Vis light conditions as illustrated in Fig. 5a, b. The experiments were done at the optimal conditions established earlier (pH 7, density 10^3^ CFU/mL and 3 g/L nanocomposite). Applying the couple of UV/g-C_3_N_4_/Fe_3_O_4_/Ag led to a complete inactivation rate for *E.Coli* and *B. subtilis*, whilst the corresponding values for the joint Vis/g-C_3_N4/Fe_3_O_4_/Ag were 68.66 and 60.53%, after 30 and 150 min, respectively (Fig. 5b).

### Effect of scavengers on inactivation of E. coli and B. subtilis

**Fig. 6:**
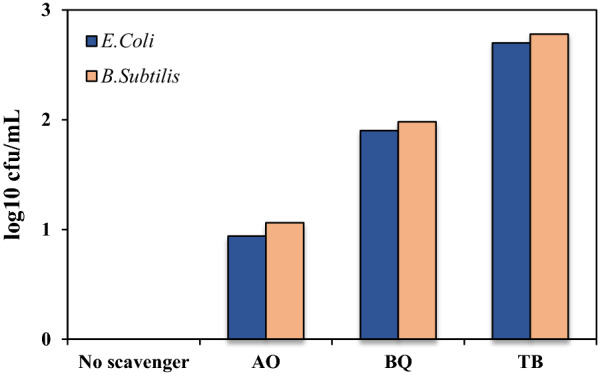
Effect of the presence of scavenger on inactivation of *E. coli* and *B. subtilis* under the optimal

Various types of reactive oxygen species (ROS) are produced during a photocatalytic process. Generally, ROS damage the cellular components such as peptidoglycan layer, electron transfer chain, bacterial genome (DNA, RNA), protein and ribosome. It also alters cell the permeability and invades the cell membrane causing it to rupture and release the cytoplasmic content (Erdem et al. 2015). In this study, the effect of three common scavengers, namely ammonium oxalate (AO), tert-butanol (TB) and benzquinone (BQ) on the inactivation of the bacteria of interest under the optimum conditions were investigated. AQ, TB and BQ were used to control the holes (h^+^), hydroxyl radicals (OH˚) and superoxide radicals (O_2_^˚−^), respectively. As depicted in Fig. 6, the rate of inactivation for both *E. coli* and *B. subtilis* was reduced in the order of AO < BQ < TB. This indicates that OH˚ is the main active species in the current photocatalytic process and the roles of h^+^ and O_2_^˚−^ are relatively negligible in this regard. Also the concentration of each scavenger was 0.1 mol.

### Kinetic models

Three kinetic models namely, the Log-Linear, Weibull and Biphasic were employed to describe the kinetics of the inactivation process at three bacterial densities of 10^3^, 10^5^ and 10^7^ CFU/mL (see Table 1). The GInaFiT software was used for the modeling, which was developed by Geeraerd et al. (2005). The statistical parameters including the coefficient of determination (R^2^) and the root mean sum of squared error (RMSE) were also used to determine the appropriate model describing the kinetics involved. Finally, a model with the maximum R^2^ and the minimum RMSE was selected as the appropriate kinetic model (Kashiri et al. 2018).

**Table 1:** Kinetic models used to describe the inactivation process

Model	Bacteria population	RMSE	R-Square	R-Square adjusted
*E. coli*				
Log-linear	10^3^	0.4043	0.9763	0.9723
	10^5^	0.4480	0.8928	0.8571
	10^7^	0.7712	0.8887	0.8510
Weibull	10^3^	0.0374	0.9998	0.9990
	10^5^	0.1368	0.9982	0.9968
	10^7^	0.1392	0.9977	0.9943
Biphasic	10^3^	0.2066	0.9948	0.9928
	10^5^	0.2624	0.9755	0.9510
	10^7^	0.2195	0.9915	0.9858
*B. subtilis*				
Log-Linear	10^3^	0.2729	0.9305	0.9450
	10^5^	0.3636	0.9547	0.9417
	10^7^	0.3624	0.9518	0.9242
Weibull	10^3^	0.1053	0.9965	0.9955
	10^5^	0.1153	0.9919	0.9878
	10^7^	0.1705	0.9905	0.9865
Biphasic	10^3^	0.1361	0.9849	0.9811
	10^5^	0.1527	0.9839	0.9806
	10^7^	0.2196	0.9825	0.9798

*RMSE* Root Mean Sum of Squared Error

### DRS analysis

**Fig. 7:**
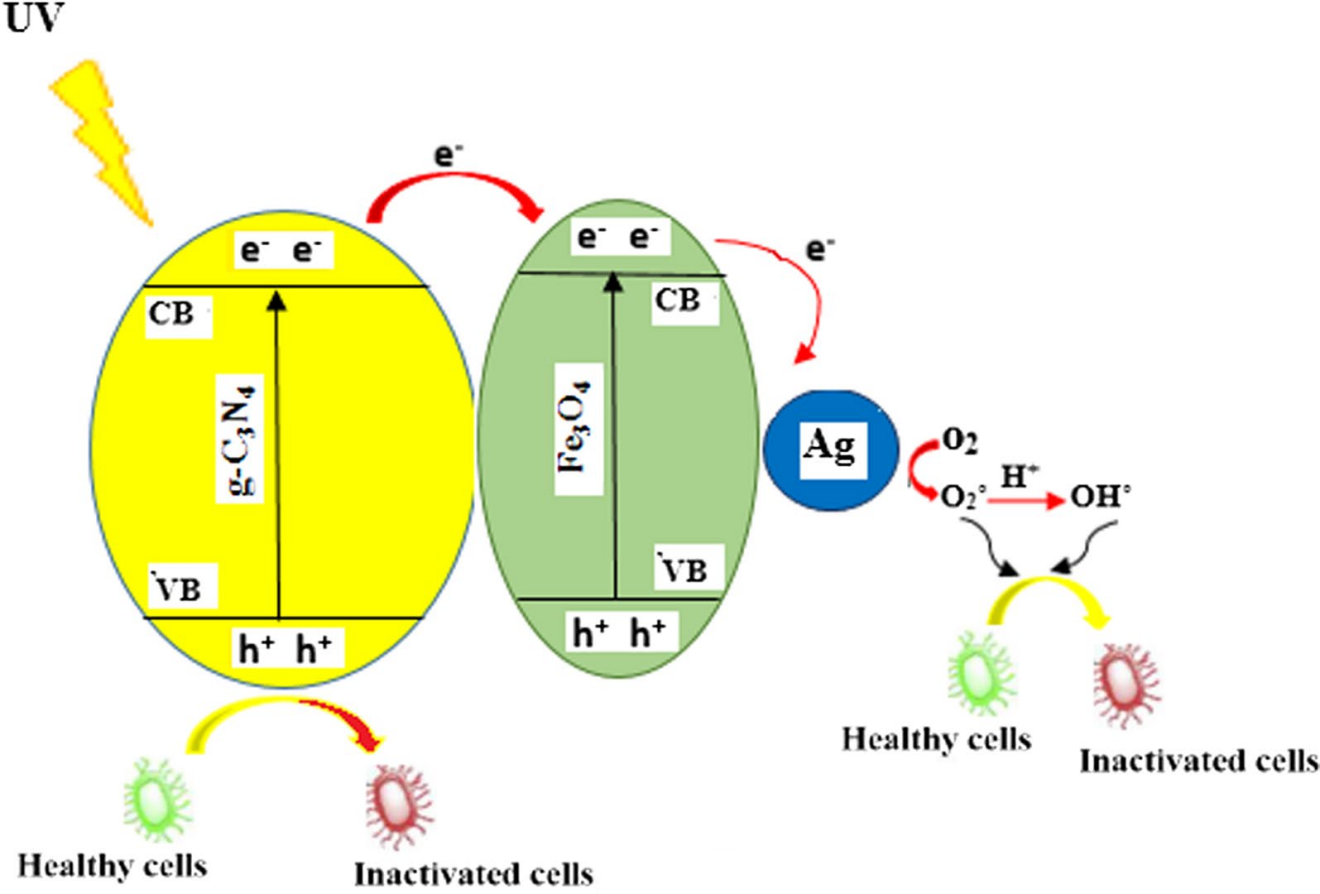
A schematic diagram representing the photocatalytic mechanism proposed for the inactivation of *E. coli* and *B. subtilis*

**Fig. 8:**
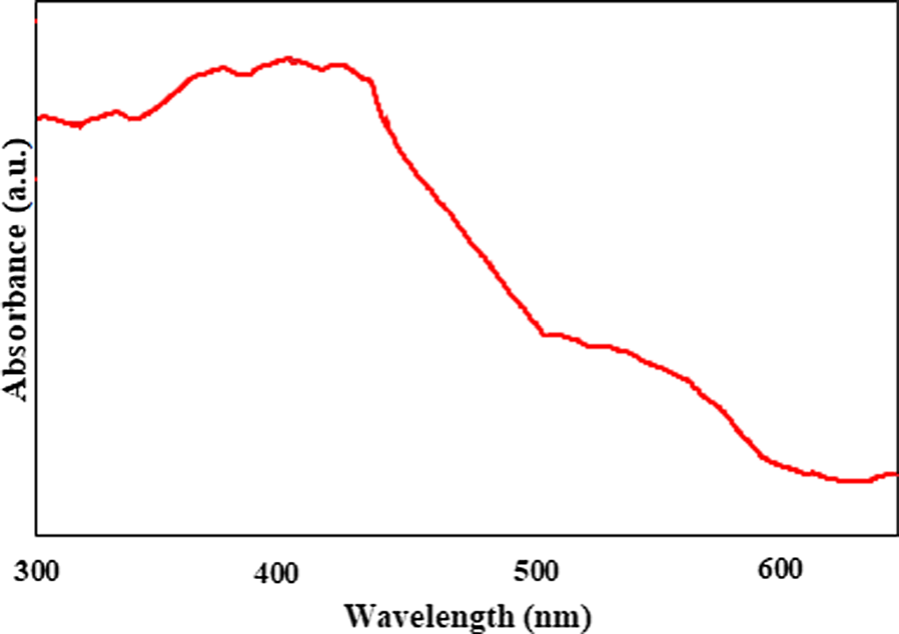
The UV–vis DRS for g-C3N4/Fe3O4/Ag

The photocatalytic activity of catalysts is closely related to their ability to absorb light. The UV–vis DRS is shown in Fig. 7 for g-C3N4/Fe3O4/Ag showed very strong absorption in the range of 400 to 700 nm. The adsorption peak is between 480 and 500 cm^-1^, which may be due to the intensification of surface plasmon resonance (SPR) of Ag species. The wider light absorption region g-C3N4/Fe3O4/Ag is able to maximize the use of light and produce load carriers by producing more efficient light, resulting in higher photocatalytic activity (Fig. 8).

## Discussion

Increasing the nanocomposite dosage leads to a shape increase in the number of photons absorbed followed by the generation of further active radicals. However, with an increase in the nanocomposite dosage from 3 to 5 g/L, the respective inactivation rate fell down to 91.8% and 87.4% for *E. coli* and *B. subtilis*. It can be justified by the reason that with an excessive rise in the dosage of nanocomposite the turbidity increases. As a result, the UV photons are prevented from reaching the active species leading to a decline in the rate of inactivation (Helali et al. 2014; Benabbou et al. 2007). *B. subtilis* shows a high level of resistance due to the presence of a thick layer of peptidoglycan around it. The main difference between gram-positive and gram-negative bacteria arise from the cell wall and the amount of peptidoglycan membrane constituents (Al-Kobaisi, 2007). On the other hand, the different responses shown to the similar dosages of nanocomposite in the gram-negative and gram-positive bacteria might be attributed to the physiological differences, intra-bacterial metabolism and selective membrane permeability, all of which are dependent on the presence/absence of light (Felczak et al. 2012). It is worth noting that the results obtained are consistent with the findings in a further related report (Alikhani et al. 2013) inactivation rates decreased can be explained by the fact that the rise in the bacterial density prevents light from penetrating into the surface of the nanocomposite followed by a sharp decline in the production of active radicals (Widi et al. 2018). On the other hand, under the constant dosage of nanocomposite, increasing the bacterial density led to a drop in the number of radicals produced resulting in reduction of the inactivation rate (Zhan et al. 2014). Our findings are in agreement with other related reports in the literature (Wang et al. 2015a).

Compared to the Vis light, the application of UV light yeilded higher inactivation rates for both bacteria the UV light activates the radicals, and destroys the cellular and enzymatic structure. Furthermore, In the dark condition, the inactivation rates for the *E. coli* were determined to be 12.6 and 21.73%, whilst the corresponding values for the *B. subtilis*, were 13.63 and 17.66%, respectively. The application of the current advanced oxidation process (AOP) using UV/g-C_3_N_4_/Fe_3_O_4_/Ag, which is accompanied with the production of OH° and further reactive agents, results in a higher bacterial inactivity than any single AOP alone. It should be mentioned that in a photocatalytic process, the UV-excited catalyst is responsible for the production of the most active radicals (Mansoury et al. 2015).

**Fig. 9:**
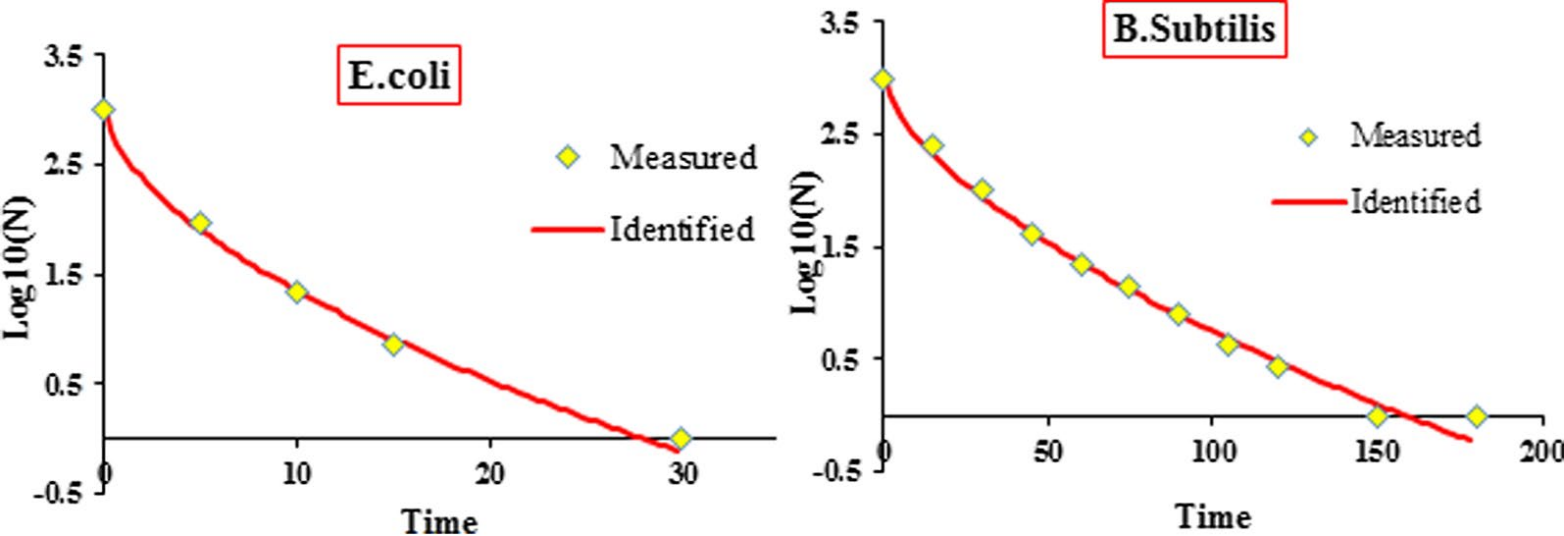
Experimentally Survival values (Measured) and the model-fit values (Identified) derived from the Weibull model for *E. coli* and *B. subtilis*

The results demonstrated that the Weibull model fitted best to the inactivation process. Compared with the other models, this model produced the lower RMSE and higher R^2^ values (Table 1). According to the Weibull model, the resistance of the individual member of population of bacteria to the inactivation process is not the same. In other words, the microbial population is of high diversity and each cell needs a specific contact time to deactivate(C Mecha et al. 2019). In a further related study, Mecha et al. reported that the Weibull model described the inactivation of the bacteria much better than the first-order kinetic model (Mecha et al. 2016). In the Weibull model, for β < 1 values an upward concave trend is observed whilst for β > 1 a downward concave trend is seen. On the other hand, the first-order kinetic is accompanied with β = 1. The results depicted in Fig. 9 reveals that the survival curve is of upward concave shape (b < 1). This implies that over the time the bacterial cells have weakened and the damage to the bacterial cells has increased (van Boekel, 2002).

### Mechanism of photocatalytic process

When g-C_3_N_4_ is exposed to light, the electrons move from the valence bond to the conduction bond, leaving a series of holes (h^+^) behind. Because Fe_3_O_4_ has a high electrical conductivity, it rapidly transfers electrons to Ag with sufficient storage capacity. It also improves the charge separation process throughout the photocatalytic system. Following that, the electrons produced react with O_2_ to produce O_2_^˚−^. The generated O_2_^˚−^ may react with h^+^ to produce OH˚ radicals. The radicals generated during the photocatalytic process (including OH) can inactivate the bacteria and subsequently damage their cells via various routs such as cell membrane destruction, inactivation of enzymes and essential proteins, and damaging DNA (Hamblin and Hasan, 2004).

## Data Availability

The dataset supporting the conclusions of this article is included within the article.

## Abbreviations

BHI: Brain Heart Infusion
VP: Poly vinyl pyrrolidone
EMB agar: Eosin Methylene-Blue agar

## References

[CR1] Abeledo-Lameiro MJ, Ares-Mazás E, Gómez-Couso H (2016). Evaluation of solar photocatalysis using TiO2 slurry in the inactivation of *Cryptosporidium parvum oocysts* in water. J Photochem Photobiol B.

[CR2] Akhundi A, Habibi-Yangjeh A (2016). Codeposition of AgI and Ag2CrO4 on g-C3N4/Fe3O4 nanocomposite: novel magnetically separable visible-light-driven photocatalysts with enhanced activity. Adv Powder Technol.

[CR3] Akhundi A, Habibi-Yangjeh A (2017). High performance magnetically recoverable g-C3N4/Fe3O4/Ag/Ag2SO3 plasmonic photocatalyst for enhanced photocatalytic degradation of water pollutants. Advanced Powder Technology.

[CR4] Albert I, Mafart P (2005). A modified Weibull model for bacterial inactivation. Int J Food Microbiol.

[CR5] Alikhani M-Y, Lee S, Yang J, Shirzad-Siboni M, Peeri-Dogaheh H, Khorasani MS, Nooshak MA, Samarghandi MR (2013). Photocatalytic removal of *Escherichia coli* from aquatic solutions using synthesized ZnO nanoparticles: a kinetic study. Water Sci Technol.

[CR6] Al-Kobaisi MF (2007). Jawetz, Melnick & Adelberg’s Medical Microbiology 24th Edition Sultan Qaboos University. Med J.

[CR7] Armon R, Weltch-Cohen G, Bettane P (2004). Disinfection of *Bacillus *spp* spores* in drinking water by TiO2 photocatalysis as a model for *Bacillus anthracis*. Water Sci Technol.

[CR8] Benabbou A, Derriche Z, Felix C, Lejeune P, Guillard C (2007). Photocatalytic inactivation of *Escherischia coli:* Effect of concentration of TiO2 and microorganism, nature, and intensity of UV irradiation. Appl Cataly B.

[CR9] Blatchley ER, Gong W-L, Rose JB, Huffman DE, Otaki M, Lisle JT (2005) Effects of wastewater disinfection on human health. Water Environment Research Foundation Alexandria, Virginia 10.2175/106143006x102024 17290975

[CR10] Cerf O (1977). A review tailing of survival curves of bacterial spores. J Appl Bacteriol.

[CR11] Di Palma L, Bavasso I, Capocelli M, De Filippis P, Piemonte V (2019). Biological treatment of wastewater from pyrolysis plant: effect of organics concentration, pH and temperature. Water.

[CR12] Ding X, Dong X, Lei J, Ding J, Ke D, Zixin Y, Shengyao W, Hao Ch (2018). Simple fabrication of Fe 3 O 4/C/gC 3 N 4 two-dimensional composite by hydrothermal carbonization approach with enhanced photocatalytic performance under visible light. Catal Sci Technol.

[CR13] Erdem A, Metzler D, Cha D, Huang C (2015). Inhibition of bacteria by photocatalytic nano-TiO 2 particles in the absence of light. Int J Environ Sci Technol.

[CR14] Fang Z, Yang J, Cao Y, Zhu L, Zhang Q, Shu D, He C (2013). Disinfection of *E. coli* using visible-light-driven photocatalyst Procedia. Environ Sci.

[CR15] Feilizadeh M, Mul G, Vossoughi M (2015). *E. coli* inactivation by visible light irradiation using a Fe–Cd/TiO2 photocatalyst: statistical analysis and optimization of operating parameters. Appl Catal B.

[CR16] Felczak A, Wronska N, Janaszewska A, Klajnert B, Bryszewska M, Appelhans D, Voit B, Rozalska S, Lisowska K (2012). Antimicrobial activity of poly (propylene imine) dendrimers. New Journal of Chemistry.

[CR105] Geeraerd A, Valdramidis V, Van Impe J (2005). GInaFiT, a freeware tool to assess non-log-linear microbial survivorcurves. Int J Food Microbiol.

[CR17] Ghodsi S, Esrafili A, Kalantary RR, Gholami M, Sobhi HR (2020). Synthesis and evaluation of the performance of g-C3N4/Fe3O4/Ag photocatalyst for the efficient removal of diazinon: kinetic studies. J Photochem Photobiol A: Chem.

[CR18] Hamblin MR, Hasan T (2004). Photodynamic therapy: a new antimicrobial approach to infectious disease?. Photochem Photobiol Sci.

[CR19] Helali S, Polo-López MI, Fernández-Ibáñez P, Ohtani B, Amano F, Malato S, Guillard C (2014). Solar photocatalysis: a green technology for *E. coli* contaminated water disinfection. Effect of Concentration and Different Types of Suspended Catalyst. J Photochem Photobiol a: Chem.

[CR20] Kashiri M, Marin C, Garzón R, Rosell CM, Rodrigo D, Martínez A (2018). Use of high hydrostatic pressure to inactivate natural contaminating microorganisms and inoculated *E. coli* O157: H7 on *Hermetia illucens* larvae. PLoS ONE.

[CR21] Li G, Nie X, Chen J, Jiang Q, An T, Wong P, Zhang H, Zhao H, Yamashita H (2015). Enhanced visible-light-driven photocatalytic inactivation of *Escherichia coli* using g-C3N4/TiO2 hybrid photocatalyst synthesized using a hydrothermal-calcination approach. Water Res.

[CR22] Li B, Meng M, Cui Y, Wu Y, Zhang Y, Dong H, Zhu Zh, Feng Y, Wu Ch (2019). Changing conventional blending photocatalytic membranes (BPMs): Focus on improving photocatalytic performance of Fe3O4/g-C3N4/PVDF membranes through magnetically induced freezing casting method. Chem Eng J.

[CR23] Ma S, Zhan S, Jia Y, Shi Q, Zhou Q (2016). Enhanced disinfection application of Ag-modified g-C3N4 composite under visible light. Appl Cataly B.

[CR24] Mafart P, Couvert O, Gaillard S, Leguérinel I (2002). On calculating sterility in thermal preservation methods: application of the Weibull frequency distribution model. Int J Food Microbiol.

[CR25] Mansoury M, Godini H, Shams Khorramabadi G (2015). Photocatalytic removal of natural organic matter from aqueous solutions using zinc oxide nanoparticles immobilized on glass. Iran J Health Environ.

[CR26] Matin AR, Yousefzadeh S, Ahmadi E, Mahvi A, Alimohammadi M, Aslani H, Nabizadeh R (2018). A comparative study of the disinfection efficacy of H2O2/ferrate and UV/H2O2/ferrate processes on inactivation of *Bacillus subtilis* spores by response surface methodology for modeling and optimization. Food Chem Toxicol.

[CR27] Mecha AC, Onyango MS, Ochieng A, Momba MN (2016). Impact of ozonation in removing organic micro-pollutants in primary and secondary municipal wastewater: effect of process parameters. Water Sci Technol.

[CR28] Mecha AC, Onyango MS, Ochieng A, Momba MN, Mecha AC, Onyango MS, Ochieng A, Momba MN (2019). Modelling inactivation kinetics of waterborne pathogens in municipal wastewater using ozone. Environ Eng Res.

[CR29] Mousavi M, Habibi-Yangjeh A (2015). Ternary g-C3N4/Fe3O4/Ag3VO4 nanocomposites: novel magnetically separable visible-light-driven photocatalysts for efficiently degradation of dye pollutants. Mater Chem Phys.

[CR30] Mousavi M, Habibi-Yangjeh A (2016). Novel magnetically separable gC 3 N 4/Fe 3 O 4/Ag 3 VO 4/Ag 2 CrO 4 nanocomposites as efficient visible-light-driven photocatalysts for degradation of water pollutants. J Mater Sci.

[CR31] Mousavi M, Habibi-Yangjeh A (2017). Novel magnetically separable g-C3N4/Fe3O4/Ag3PO4/Co3O4 nanocomposites: visible-light-driven photocatalysts with highly enhanced activity. Adv Powder Technol.

[CR32] Ouyang K, Dai K, Walker SL, Huang Q, Yin X, Cai P (2016). Efficient photocatalytic disinfection of *Escherichia coli* O157: H7 using C 70-TiO 2 hybrid under visible light irradiation. Sci Rep.

[CR33] Pant B, Pant HR, Barakat NA, Park M, Han T-H, Lim BH, Kim H-Y (2014). Incorporation of cadmium sulfide nanoparticles on the cadmium titanate nanofibers for enhanced organic dye degradation and hydrogen release. Ceram Int.

[CR34] Pant B, Park M, Lee JH, Kim H-Y, Park S-J (2017). Novel magnetically separable silver-iron oxide nanoparticles decorated graphitic carbon nitride nano-sheets: a multifunctional photocatalyst via one-step hydrothermal process. J Colloid Interface Sci.

[CR35] Rojviroon T, Sirivithayapakorn S (2018) *E. coli* bacteriostatic action using TiO2 photocatalytic reactions. Int J Photoenergy

[CR36] Ruales-Lonfat C, Barona JF, Sienkiewicz A, Vélez J, Benítez LN, Pulgarín C (2016). Bacterial inactivation with iron citrate complex: a new source of dissolved iron in solar photo-Fenton process at near-neutral and alkaline pH. Appl Catal B.

[CR37] Sondi I, Salopek-Sondi B (2004). Silver nanoparticles as antimicrobial agent: a case study on *E. coli* as a model for Gram-negative bacteria. J Colloid Interface Sci.

[CR38] Spuhler D, Rengifo-Herrera JA, Pulgarin C (2010). The effect of Fe2+, Fe3+, H2O2 and the photo-Fenton reagent at near neutral pH on the solar disinfection (SODIS) at low temperatures of water containing *Escherichia coli* K12. Applied Catalysis B: Environmental.

[CR39] Sun J-H, Sun S-P, Fan M-H, Guo H-Q, Qiao L-P, Sun R-X (2007). A kinetic study on the degradation of p-nitroaniline by Fenton oxidation process. J Hazard Mater.

[CR40] Tran QH, Le A-T (2013). Silver nanoparticles: synthesis, properties, toxicology, applications and perspectives. Adv Nat Sci.

[CR41] van Boekel MA (2002). On the use of the Weibull model to describe thermal inactivation of microbial vegetative cells. Int J Food Microbiol.

[CR42] Wang M, Cui S, Yang X, Bi W (2015). Synthesis of g-C3N4/Fe3O4 nanocomposites and application as a new sorbent for solid phase extraction of polycyclic aromatic hydrocarbons in water samples. Talanta.

[CR43] Wang W, Huang G, Jimmy CY, Wong PK (2015). Advances in photocatalytic disinfection of bacteria: development of photocatalysts and mechanisms. J Environ Sci.

[CR44] Widi RK, Savitri E, Angelina O, Caroline S, Budhyantoro A (2018). Antibacterial inactivation of *Escherichia coli* after TiO2-Fe3O4-bentonite photocatalytic treatment international journal on advanced science. Eng Inform Technol.

[CR45] Xia D, Wang W, Yin R, Jiang Zh, An T, Li G, Zhao H, Wong P (2017). Enhanced photocatalytic inactivation of *Escherichia coli* by a novel Z-scheme g-C3N4/m-Bi2O4 hybrid photocatalyst under visible light: the role of reactive oxygen species. Appl Cataly B.

[CR46] Zazouli MA, Yousefi M, Kor Y, Roohafzaee M (2017). Inactivation of *Escherichia coli* in water by combined process of silver nanoparticle and ultraviolet radiation. Health Scope.

[CR47] Zhan S, Yang Y, Shen Z, Shan J, Li Y, Yang S, Zhu D (2014). Efficient removal of pathogenic bacteria and viruses by multifunctional amine-modified magnetic nanoparticles. J Hazard Mater.

[CR48] Zhang Y, Zhou L, Zhang Y (2014). Investigation of UV–TiO2 photocatalysis and its mechanism in *Bacillus subtilis spore* inactivation. J Environ Sci.

[CR49] Zhang X, Wu Y, Xiao G, Tang Z, Wang M, Liu F, Zhu X (2017). Simultaneous photocatalytic and microbial degradation of dye-containing wastewater by a novel g-C3N4-P25/photosynthetic bacteria composite. PLoS ONE.

[CR50] Zhu Z, Lu Z, Wang D, Tang X, Yan Y, Shi W, Wang Y, Gao N, Yao X, Dong H (2016). Construction of high-dispersed Ag/Fe3O4/g-C3N4 photocatalyst by selective photo-deposition and improved photocatalytic activity. Appl Cataly B.

[CR51] Zhu Z, Yu Y, Dong H, Liu Z, Li C, Huo P, Yan Y (2017). Intercalation effect of attapulgite in g-C3N4 modified with Fe3O4 quantum dots to enhance photocatalytic activity for removing 2-mercaptobenzothiazole under visible light. ACS Sustain Chem Eng.

